# Research on the printability of hydrogels in 3D bioprinting

**DOI:** 10.1038/srep29977

**Published:** 2016-07-20

**Authors:** Yong He, FeiFei Yang, HaiMing Zhao, Qing Gao, Bing Xia, JianZhong Fu

**Affiliations:** 1 State Key Laboratory of Fluid Power and Mechatronic Systems, College of Mechanical Engineering, Zhejiang University, Hangzhou 310027, China; 2 Key Laboratory of 3D Printing Process and Equipment of Zhejiang Province, College of Mechanical Engineering, Zhejiang University, Hangzhou 310027, China; 3 State Key Laboratory for Manufacturing Systems Engineering, Xi’an Jiaotong University, 710054, Xi’an China

## Abstract

As the biocompatible materials, hydrogels have been widely used in three- dimensional (3D) bioprinting/organ printing to load cell for tissue engineering. It is important to precisely control hydrogels deposition during printing the mimic organ structures. However, the printability of hydrogels about printing parameters is seldom addressed. In this paper, we systemically investigated the printability of hydrogels from printing lines (one dimensional, 1D structures) to printing lattices/films (two dimensional, 2D structures) and printing 3D structures with a special attention to the accurate printing. After a series of experiments, we discovered the relationships between the important factors such as air pressure, feedrate, or even printing distance and the printing quality of the expected structures. Dumbbell shape was observed in the lattice structures printing due to the hydrogel diffuses at the intersection. Collapses and fusion of adjacent layer would result in the error accumulation at *Z* direction which was an important fact that could cause printing failure. Finally, we successfully demonstrated a 3D printing hydrogel scaffold through harmonize with all the parameters. The cell viability after printing was compared with the casting and the results showed that our bioprinting method almost had no extra damage to the cells.

3D printing (additive manufacturing) is now widely applied in the electronics, automotive, aerospace, medical engineering and other fields^1^. Due to high precision, high efficiency for single product and convenient operation, 3D printing gets more and more attention from the whole word. In recent years, this technology has been widely used in the tissue engineering. The term coined for the printing of tissues using these types of approaches is bioprinting, cell printing or even organ printing^2^. Bioprinting uses a computer-controlled 3D printing device to accurately deposit cells and biomaterials into the models of organs^3^. It has less danger of organ transplant rejection than traditional treatment of grafting, which is limited by the number of donors. Nowadays several bioprinting progresses have been demonstrated, such as bionic ears^4^, multilayered skins^5^, artificial bones^6^, vascular tissues^7^ and cartilaginous structures^8^.

Hydrogels come out as a kind of biomaterial with good biocompatibility and now they are widely used as the cell-laden materials for bioprinting. A number of bioprinting methods have been explored, including cellular inkjet printing^9^,^10^,^11^,^12^,^13^,^14^, laser-assisted bioprinting^15^,^16^,^17^,^18^, stereolithography^19^,^20^,^21^ and extrusion-based printing^22^,^23^,^24^. Inkjet printing owns high printing speed, low cost and wide availability, but has the risk of exposing cells to thermal & mechanical stress and unreliable cell encapsulation. Laser-assisted bioprinting represents a promising method owing to its fine resolution while the high cost of printing systems is a concern. Also, lack of commercial 3D laser bioprinters will limit its wide use. Stereolithography is known for high accuracy, but its disadvantages include lack of printing multi-cells and the damage of cell during photocuring. Although the pressure of extrusion-based bioprinting may have the effect on cell viability, it is a common method for the ability to deposit very high cell densities. Currently, the extrusion-based printer equipped with two printing heads had printed tubular constructs^25^. Some groups achieved fabrication and scale-up of 3D structures by the use of viscous biomaterials and extrusion-based technologies^26^,^27^,^28^. “Scaffold-free” bioprinting based on extrusion-based printing has been coined, which follows the principles of tissue liquidity and tissue fusion of multi-cellular components^7^. Multicellular cell spheroids are deposited and allowed to self-assemble into the desired 3D structure^29^,^30^. Recently, we also demonstrated that micro channels used for nutrients delivery as vessels in the printed tissues could be printed with the 3D hydrogel/cell structures at the same time with coaxial nozzle-assisted bioprinting by extrusion^31^.

With precisely controlled deposition of cell-laden hydrogels, organs can be mimicked better as the 3D structures determine nurture morphology and growth characteristics of cells after printing. It is clear that the 3D hydrogel printability study is very important in tissue engineering. In the research of hydrogel printability, many investigations have been performed including, assessing the printability of the ink solutions using rheology and ink consistency^23^; discussing physical and rheological properties of hydrogel under the conditions imparted by different biofabrication instruments^32^; finding a direct correlation between printability and the hydrogel mechanical properties^33^. However, little attention has so far been paid to various process parameters during printing and discussing the relationships between the parameters and the printing fidelity. There are many parameters which will have great influences on printing resolution during the extrusion-based bioprinting process, such as the hydrogel fusion during printing, deformation caused by gravity, non-uniform extrusion due to the change of printing speed. It is short of research reports about systematically discussing the printability of biomaterials or the relationships between printing quality/fidelity and the process parameters.

In this paper, the hydrogel was the mixture of sodium alginate (SA) and gelatin with a proper rate. The mixture was extruded on a cool substrate for the solidification of gelatin and fixing the biostructures. After printing, the structures were immersed in the calcium chloride (CaCl_2_) solution for the crosslinking of SA and acquiring the cell-laden hydrogel structures. The influence of air pressure, feedrate, printing distance, and printing sharp angle were discussed separately in printing lines (1D structures) as lines were the base units of 2D and 3D structures. Next we systemically discussed the fidelity of lattice structures (2D structures) and 3D structures, in order to reduce the diffusion and collapse during printing. Finally, we successfully demonstrated a 3D printed hydrogel structure through harmonizing all the parameters.

## Results

### Bioprinting of 3D structures

**Figure 1:**
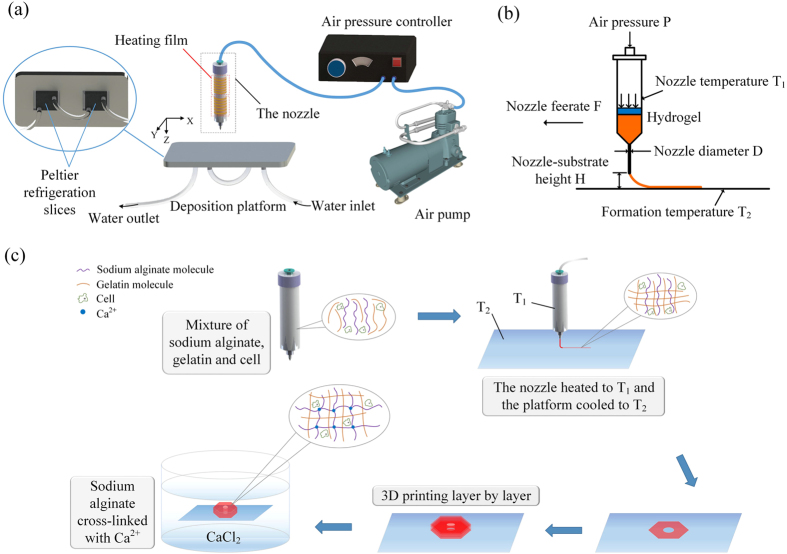
Schematic showing of the bioprinting system. **(a)** Diagram of the 3D bioprinting system, heating film on the nozzle was used to enhance the flow behavior of bioink, peltier refrigeration was used to keep cool and fix the bioink; **(b)** Printing parameters of this system; (**c**) Schema of the printing process. The structure is built layer by layer, and when finished it is immersed in the solution of calcium chloride. Figure 1 was drawn by FeiFei Yang.

Our self-designed 3D bioprinter includes a *XYZ* moving platform with a cooling substrate (Peltier cooler) to contain the printed hydrogels and a nozzle could be heated to extrude hydrogel easily under air pressure, as shown in Fig. 1a. The hydrogel precursor used in experiments is SA, which is widely used in biofabrication^34^,^35^,^36^,^37^. For better printing quality, gelatin was mixed and was used to hold the sharp of printing structures before SA was crosslinked to hydrogel. For the simplicity, the sol (mixture of SA/gelatin/water) used before printing is also called hydrogel although it is not crosslinked. Currently, some literatures about the matrix of alginate/gelatin with enhanced properties are reported^38^,^39^,^40^. These researches are mainly concerned about improving the mechanical or biocompatible performance of the mixture. As cell-laden is not required in the scaffold fabricating, interpenetrating polymer networks (IPN) can be realized with some additional cross-linking agents or processing such as freeze-drying. The fundamental purpose of gelatin used in this report is for keeping the shape of the printed structures as it will be gelled when temperature is lower than its solidification temperature.

During printing, suitable viscosity is very important. Deformation and collapse will be easily happened when printing low viscosity materials. On the other hand, nozzles will be easily jammed when high viscosity materials are printed. To avoid these problems, as shown in Fig. 1b, firstly, the mixture was heated on the nozzle with a temperature *T*_1_, then it was extruded under air pressure to the substrate and cooled to a temperature *T*_2_ with two thermoelectric coolers. *T*_1_ must be higher than the melting temperature of gelatin to avoid jamming nozzle. *T*_2_ must be lower than the solidification temperature of gelatin to fix the printed structures. Desiccant was embedded in the substrate in order to decrease the influence of condensed moisture. The substrate was designed to move up/down as the *Z* direction in order to avoid vibration. After printing, the whole structures were dropped in the CaCl_2_ solution to crosslink SA. The whole fabrication process for a 3D structure is shown in Fig. 1c.

### Material ratio of the hydrogel

According to many experiments, we found the viscosity of extruding material, *η*, should be lower than 100000 cps. The viscosity is suitable at the range of 300–30000 cps (ln*η* = 5.70–10.31). Material with a viscosity below 300 cps is more suitable for smearing instead of printing. While when the viscosity is higher than 30000 cps, large pressure is needed to extrude the hydrogel out of the nozzle, and what’s more the extrusion process will become unstable. From the Table 1, the suitable printing concentration of SA is in the range of 2–4%.

Furthermore, five control groups were prepared in the experiments. Each group was produced with fixed amounts of SA and varying gelatin concentrations. As shown in Table 2, according to the above analysis about suitable viscosity, it is better that the mixture has a concentration of SA in the range of 2–2.5% and gelatin in the range of 4–8%. A mixture of 2.5% SA and 8% gelatin was used in the following experiments.

Some tensile testing were performed to see was there any strength difference with/without gelatin after the SA was cross-linked. As shown in Fig. S1a, the tensile strength of gelatin soaked in calcium chloride solution was lower than the gelatin without any treatment. Researches about increasing mechanical strength of gelatin hydrogel also found that the removal of divalent ions can significantly improve the stability and strength of gelatin hydrogel^41^,^42^. However, the impact of Ca^2+^ for gelatin was tiny because the gelatin polymer network is highly hydrophilic, which absorbs water through hydrogen bonds formed between water molecules and carboxylic acid and amino groups^41^. That’s why the tensile strength of sodium alginate/gelatin hydrogel (crosslinked with CaCl_2_) is higher than sodium alginate hydrogel (crosslinked with CaCl_2_), as shown in Fig. S1b.

When gelatin is mixed with sodium alginate and heated to 37 °C, sodium ion had a weak electrostatic interaction with COO-, leading to freer carboxylic acid groups. Then the mixture is cooled on the deposition platform while printing, and the gelatin network adsorbs water through hydrogel bonds. The electrostatic interaction between calcium ion and COO- is weaker than the interaction between hydrogen bonds and COO-41. Only a little calcium ion is reacted with gelatin molecule. Duan *et al*. also reported that gelatin was gradually released from ionically crosslinked hydrogel discs^36^. So there is rather an interaction force than a cross-linking force between gelatin and CaCl_2_ after printing.

### Line printing

**Figure 2:**
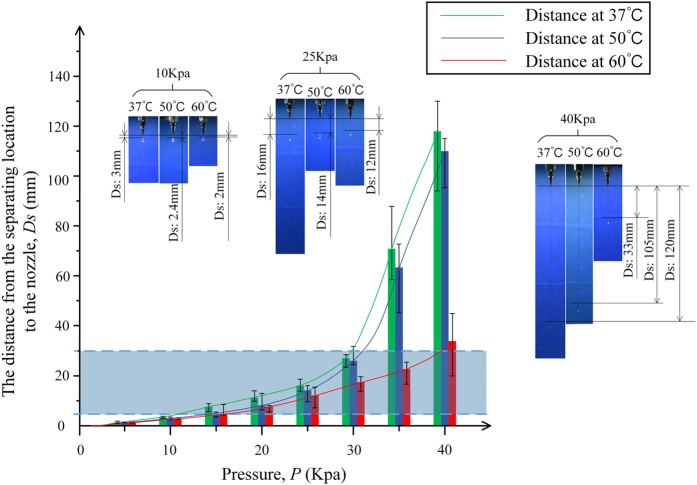
The influence of air pressure on the printability of hydrogel. *D* = 0.4 mm. The experiment was repeated at least five times and the standard deviation was less than 5% shown by error bars.

The air pressure, *P*, is the most important parameter of all since it determines the extrusion output, which directly affects the printed line width. *P* should be larger than the surface tension of extrusion materials in the nozzle. *P* is mainly determined by the viscosity of printing materials. Here we defined a parameter, *D*_s_, the distance from the separating location to the nozzle to denote the influence of surface tension and air pressure, which increased with the increase of air pressure. The extrusion under different pressure and different viscosity was investigated, as shown in Fig. 2. Hydrogel cannot be pushed out unless *P* > 5 KPa. Over high air pressure (when *P* > 35 KPa) would cause quick extrusion and even unstable extrusion like jetting, which means it is hard to control the extrusion and results in low printing quality. After a serial experiments, we suggest that a suitable *P* can be chosen when *D*_s_ is in the range of 5–30 mm, in other word, for the viscosity in our experiments, *P* can be chosen in the range of 15–30 KPa. As for biomaterials with different viscosity, researchers can do a simple experiment to acquire the relationship between *D*_s_ and *P*, just like the Fig. 2 and then the suitable *P* can be chosen.

**Figure 3:**
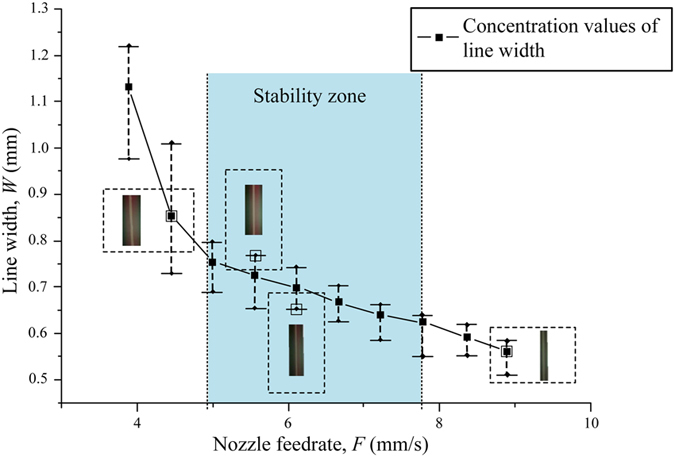
The influence of nozzle feedrate on line width. *P* = 20 KPa, *D* = 0.4 mm. The experiment was repeated at least three times and the standard deviation was less than the symbol size expect where noted by error bars.

The nozzle feedrate, *F*, affects the line width, *W*, directly. As shown in Fig. 3, the line width decreased with the increase of the nozzle federate, with *P* = 20 KPa and the nozzle diameter, *D* = 0.4 mm. The line width was more than the nozzle diameter, because of expansion after the extrusion (*F* = 4–10 mm/s). If the feedrate increasing, the line will be stretched and become thinner and thinner until the lines are broken. It is very important that the feedrate should match the air pressure.

**Figure 4:**
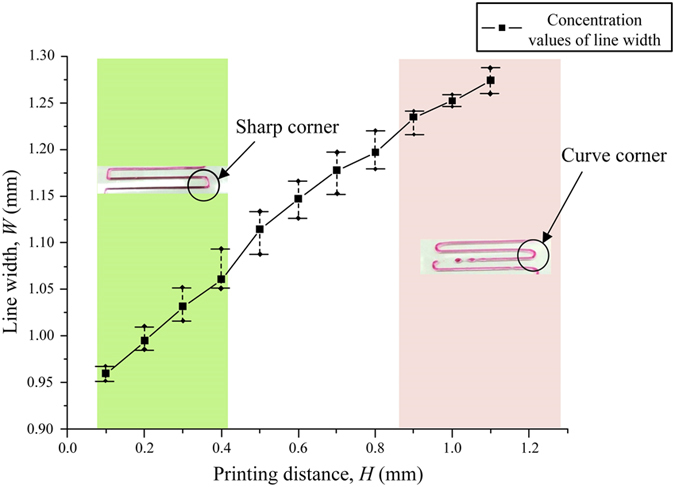
The influence of distance between nozzle and substrate on line width. *P* = 20 KPa, *D* = 0.5 mm, *F* = 4.45 mm/s. The experiment was repeated at least three times. Standard deviations greater than 3% shown by error bars.

The influence of the printing distance on the line width is shown in Fig. 4, where *H* is the distance from the nozzle to the printing platform (printing distance). With the increase of *H*, the line width also increased and they kept a nearly linear relationship (the R-squared is about 0.3). When *H* < 0.4 mm, printed lines with right angle shape were easily found, while under the same conditions of others, curve corners were observed when *H* > 0.9 mm. The reason is that the mixture of SA and gelatin is so soft, and it has a response lag when the nozzle changing direction in the printing. This response lag increased with the increasement of *H*.

### Sharp angle printing

**Figure 5:**
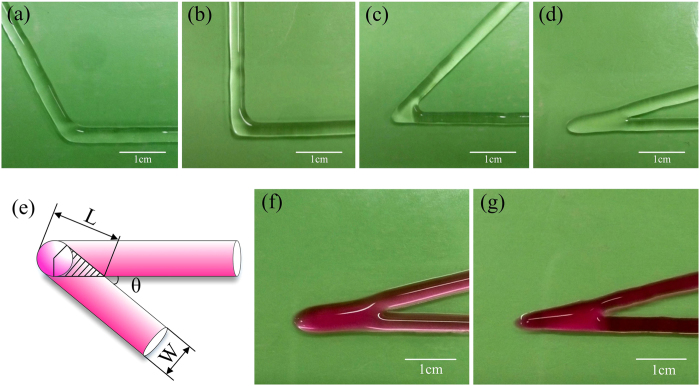
Overlap in sharp corner printing. *P* = 20 KPa, *D* = 0.4 mm, *F* = 4.45 mm/s, *H* = 0.4 mm. **(a)** Line shape of acute angle printing; **(b)** Line shape of right angle printing; **(c)** Line shape of obtuse angle printing; **(d)** Line shape of sharp angle printing; **(e)** Schema of overlap; **(f)** Double extrusion at overlap area; **(g)** Uniform extrusion by doubling *F* in the overlap area.

There is an overlap problem in sharp angle printing. Figure 5a–d shows printing the line shape with acute angle, right angle and obtuse angle at the same parameters. Printing quality became worst in acute angle. As shown in Fig. 5f, some areas were overlapped, where the extrusion of hydrogel was doubled, which would cause the non-uniform layer height in 3D structure printing. The error of layer height will be accumulated layer by layer until the failure of printing.

Two strategies can be used to avoid the non-uniform extrusion. The first method is avoiding the sharp angle in the printing path generation. However the sharp line could not be avoided when printing sharp structures. The second method is reducing the extrusion rate in this area from the normal extrusion to the half extrusion. If the nozzle is extruded by a motor, the extrusion rate can be easily controlled by the motor speed. The overlap (Fig. 5f) can be released with double moving speed (Fig. 5g).

### Lattice scaffold printing

**Figure 6:**
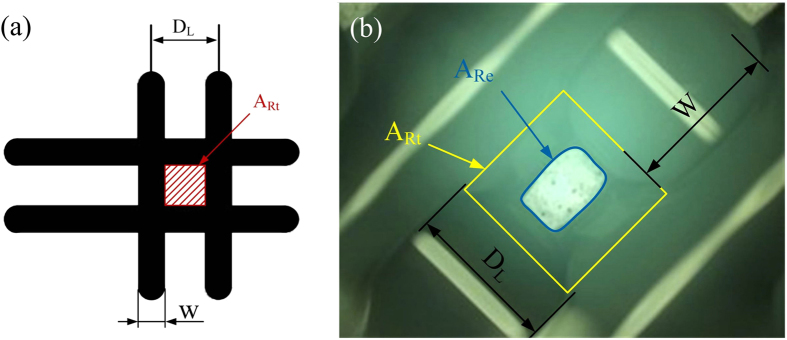
Lattice scaffold printing. **(a)** Area of a lattice in theory; **(b)** Area of a lattice in experiments. *P* = 20 KPa; *H* = 0.2 mm *F* = 4.45 mm/s.

**Figure 7:**
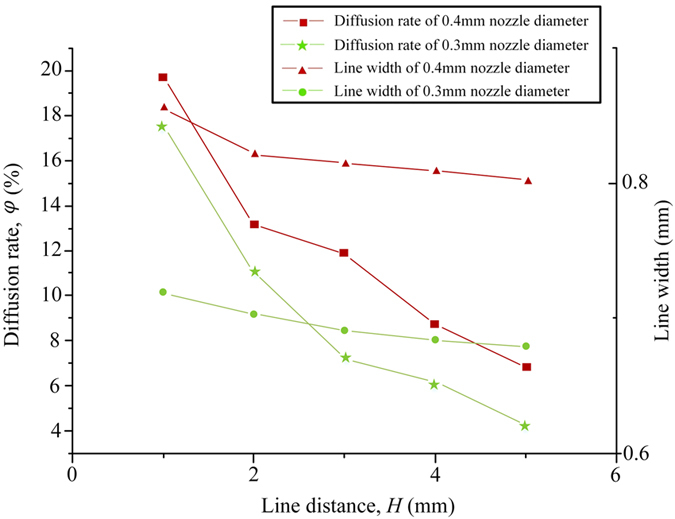
The relationship between *D*_L_ (line distance) and (diffusion rate). *P* = 20 KPa, *H* = 0.4 mm *F* = 4.45 mm/s, recorded the minimum line width.

Lattice structures are widely used in each type of cell-laden scaffolds, so it is important to precisely control the printing quality. As discussed in the line printing above, feedrate, printing distance and air pressure affected the lattice printing quality. Besides these parameters, we found line distance and intersection area of line affecting the lattice quality as well. A variable of diffusion rate can be calculated as following:


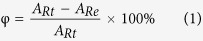


where *A*_*Rt*_ is the area of a lattice in theory and *A*_*Re*_ is the area of a lattice in experiments. As shown in Fig. 6a, with two groups of adjacent straight lines, rectangles were formed, where *D*_*L*_ is the line distance in the lattice. Theoretically, the area of a lattice *A*_*Rt*_ can be described easily from the rectangle area. While the area of the lattice in experiments *A*_*Re*_, as shown in Fig. 6b, it can be described as quadratic curves. Under microscopic observation we found that *A*_*Re*_ was much smaller than *A*_*Rt*_, and the area in experiments became irregular. In order to analyze the relationship between the line distance and the diffusion rate, a serial of lattices with different line distance from 1 mm, 2 mm… to 5 mm were printed and the diffusion rate were tested, as shwon in Fig. 7. With the increase of line distance, the diffusion rate dropped quickly. And when the line distance was large than 4 mm, the diffusion rate was not obvious. Also we found that with *D*_*L*_ increasing, *W*_min_ decreased.

**Figure 8:**
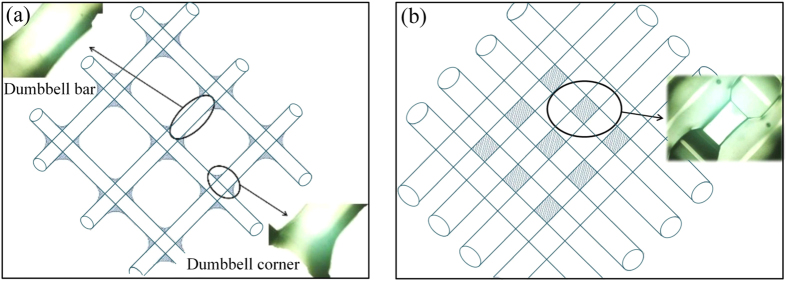
Hydrogel diffusion in the intersection. (**a)** The model and the practical shape of the dumbbell structure when *D*_L_ = 5 mm; **(b)** The model and the practical shape of the dumbbell structure when *D*_L_ = 2 mm.

At the intersection, overlapped hydrogels diffused due to the gravity. That is the reason why *A*_*Rt*_ is less than *A*_*Re*_. Dumbbell shape will be observed in the lattice structures printing, as shown in Fig. 8. The diffused hydrogel at the intersection is taken as the dumbbell corner and the straight line between the two dumbbell corners is taken as the dumbbell bar. When *D*_*L*_ = 5 mm, the actual shape coincided basically with the shape of the theoretical analysis of the dumbbell structure. The width of the dumbbell bar recovers the initial value gradually outside the intersection. However, when *D*_*L*_ = 2 mm, it can be found out clearly from the black dash line that the width of the dumbbell bar in experiments is much wider than the theory. That is because the two dumbbell corners would become very close or even overlapped with each other when *D*_*L*_ is small enough. It leads to the diffusion between two adjacent intersection and then has an effect on area overlapping. At last, hydrogel from two adjacent dumbbell corners spread and increase the line width.

**Figure 9:**
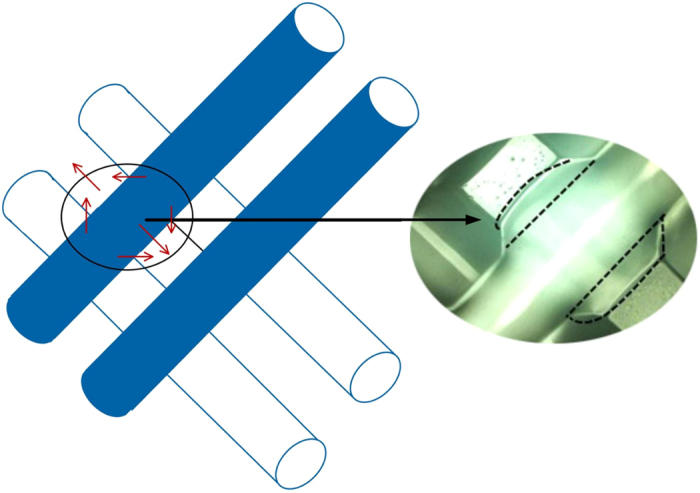
Directional diffusion.

**Figure 10:**
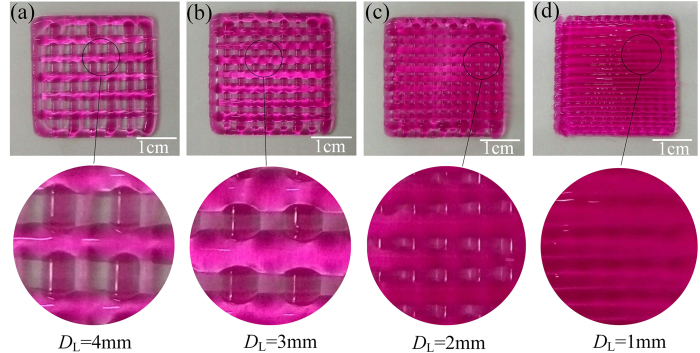
Hydrogel diffusion and fusion. **(a)** Lattice scaffold with *D*_L_ = 4 mm; **(b)** Lattice scaffold with *D*_L_ = 3 mm; **(c)** Lattice scaffold with *D*_L_ = 2 mm; **(d)** Lattice scaffold with *D*_L_ = 1 mm.

During the printing of 2D reticular structure, after finishing the first layer, the second layer would suffer squeeze and radial diffusion under the influence of the first hydrogel layer, as shown in Fig. 9. However the first layer would not be affected. Due to the diffusion phenomenon, the pore would radially narrow along the second hydrogel layer line and would not change axially. So the pores in printing hydrogel reticular structure appeared rectangular distribution, while it should be square in theory. With *D*_L_ increasing, diffusion and fusion would be mitigated, as shown in Fig. 10.

### 3D structures printing

**Figure 11:**
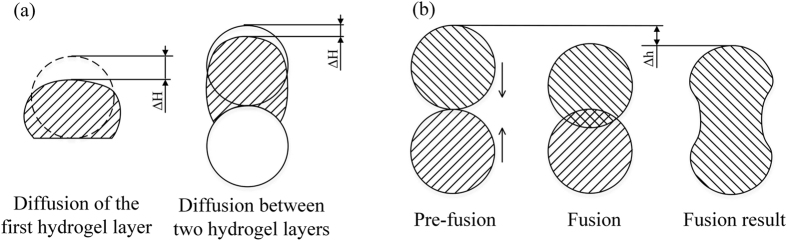
Comparison of two kinds of deformation of hydrogel profile. **(a)** Diffusion of the first hydrogel layer and two hydrogel layers; **(b)** Fusion progress of two hydrogel layers in vertically.

For the 3D printed structures, layer height cannot be decided by the printed line height in two-dimension. It should be calibrated separately and recorded into printing parameters. The width of printed-out line is bigger than the theoretical value calculated from the diameter of nozzle. While the height of collapsed line is smaller than the theoretical value. The schema of the Δ*H* decrease is shown in Fig. 11a, which is caused by the hydrogel collapses or diffusion because of gravity.

Except collapses, fusion of hydrogel can also affect printing resolution in 3D printing progress. In vertical, the two hydrogel layers fuse together and the sizes of fusion area decide the value of height difference Δ*h*. Δ*h* is caused by the fusion of two adjacent hydrogel layers as shown in Fig. 11b. According to a serial of experiments, the value of Δ*h* is much larger than Δ*H* in 3D structures. The fusion are affected by the the printing time between adjacent layers and cooling temperature *T*_2_, in other word, different structures may cause different fusion height. If a clinic size is printed, the layer height error caused by collapse and fusion should be considered.

**Figure 12:**
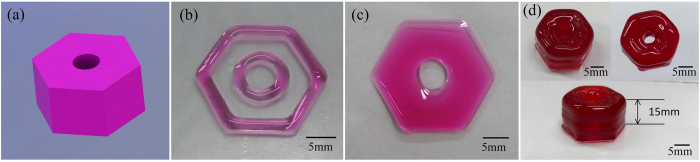
The printability of 3D printing hydrogel scaffold. **(a)** The digital model of the 3D scaffold; **(b)** The outline of the first layer of hydrogel scaffold (the line width is about 2 mm); **(c)** The first layer of scaffold shape after filled the blank area (the filling rate is 100%); **(d)** Three view of printed hydrogel scaffold form three different angle (the structure is total 30 layers and the height of it is 15 mm, the fidelity of the top surface is about 80%).

Here, we gave an example of 3D printing hydrogel scaffold as shown in Fig. 12. The ink had a concentration of 2.5%(w/v) SA and 8%(w/v) gelatin. The nozzle was heated and kept in 37 °C and the substrate was cooled to 5 °C. The parameters in printing process was set as: *P* = 20 KPa, *D* = 0.3 mm, *H* = 0.1 mm, *F* = 4.45 mm/s. The line width was 0.6 mm in theory while 0.65 mm in the experiments because of diffusion. This structure had 30 layers with a layer height of 0.57 mm. High fidelity was kept during printing previous 25 layers (Fig. 12b–d). As for the tilted surface in digital model, angle correction was made less than 30° before printing. To make sure to achieve a smaller horizontal section and decrease fusion between every two layers, the printing time of every layer was delayed larger than 40 s. After printing, 2% CaCl_2_ solution was used to solidify the 3D hydrogel scaffold. Because alginate and calcium solutions are combined, and through ionic crosslinking a gel is formed.

### Cell viability analysis

As the residual CaCl_2_ after cross-linking may affect the cell viability, calcium ion concentration was test, which was shown in Fig. S2. We found the concentration of Ca^2+^ in cell culture medium increased a lot after 24 h. However, it became stable at the concentration of 20 mg/L in the following three days. In normal cells, the concentration of dissociative Ca^2+^ is 0.1 μmol/L~1.0 μmol/L inside the cell membrane. As the cell membrane is the barrier for the flow of Ca^2+^, the concentration of dissociative Ca^2+^ outside of the cell membrane can be about 60 mg/L. So the concentration of residual Ca^2+^ (20 mg/L) after printing is still in the range of safety and will not cause extra damages to the cells.

**Figure 13:**
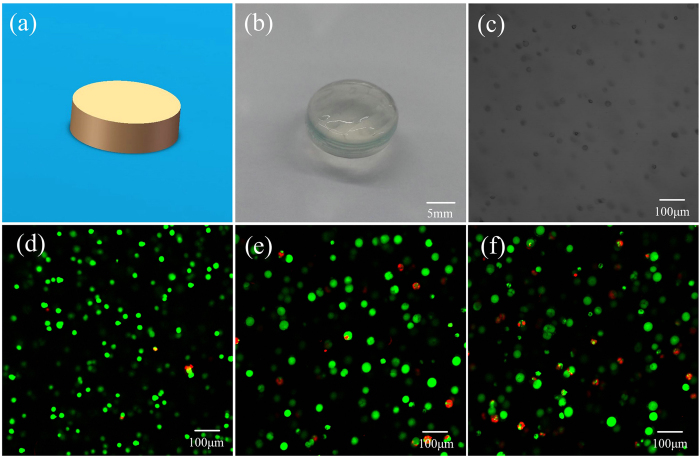
Viability of L929 mouse fibroblasts cultured in hydrogel-conditioned media in 3D printing method. **(a)** The digital model of the 3D structure; **(b)** 3D hydrogel structure fabricated by bioprinting; **(c)** Images show cells encapsulated in hydrogel uniformly; **(d–f)** Microscopic images showing the viability of cells in 1 day, 4 days and 7 days, respectively. (Live and dead cells were fluorescent green and fluorescent red, respectively).

We chose a small cylinder (diameter 10 mm, thickness 3 mm) as a typical 3D structure to examine cell analysis, just as shown in Fig. 13a. L929 mouse fibroblasts were encapsulated in hydrogel uniformly (Fig. 13b). Figure 13c–e showed cells images after staining using a laser scanning confocal fluorescence microscopy (LSCM). It could be find that more than 90% cells were alive (green) after one day (Fig. 13c). Then the rate of cell viability got down as time increased. But there were still about 60% cells alive after seven days (Fig. 13e), it indicated the biocompatibility of the hydrogel. In order to demonstrate that whether the extrusion pressure was harmful to the cell viability, the controlled group with casting method was also performed.

**Figure 14:**
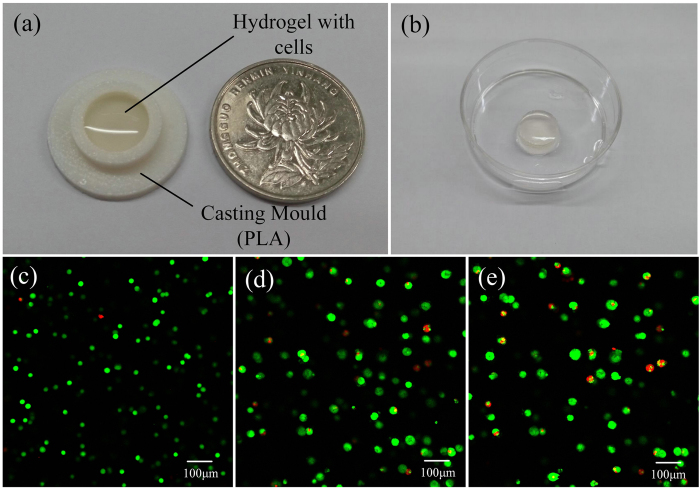
Viability of L929 mouse fibroblasts cultured in hydrogel-conditioned media in casting method. **(a)** Casting mold; **(b)** 3D hydrogel structure fabricated by casting; **(c–e)** Microscopic images showing the viability of cells in 1 day, 4 days and 7 days, respectively. (Live and dead cells were fluorescent green and fluorescent red, respectively).

As shown in Fig. 14, a cylinder with cell-laden hydrogel (2.5% SA, 8% gelatin and the cell density of 1 × 10^6^ cells/ml) was casted by pouring the bio-ink into the mold. Then it was put into 2% CaCl_2_ solution for crosslinking. Figure 14c–e shows the images of cell viability after casting. It indicated the biocompatibility of the hydrogel and the extrusion pressure had little influence on the cells.

**Figure 15:**
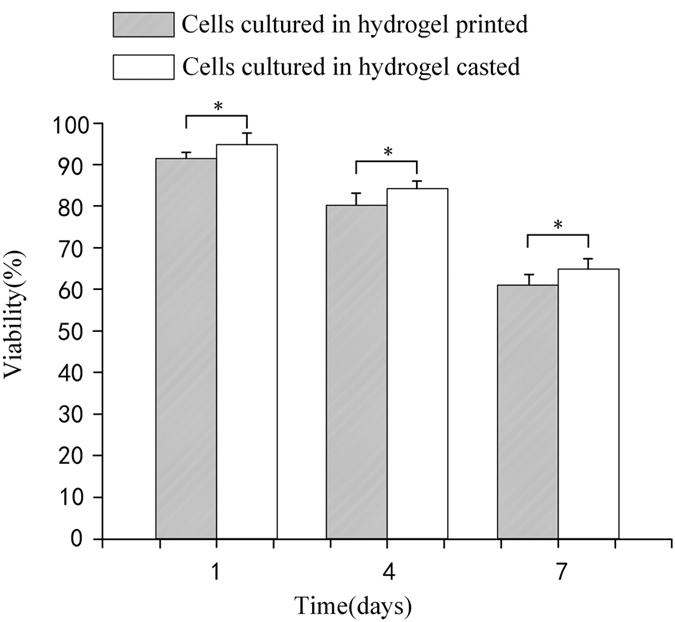
Viability of L929 mouse fibroblasts cultured in hydrogel-conditioned media over 7 days’ period. The error bars show mean ± SD of independent replicates, single asterisk (*) indicates significant differences between groups (p < 0.05).

Figure 15 shows the viability percentage of cells in hydrogel-conditioned media fabricated both by printing and casting. For the printed cell-laden hydrogel structure, the cell viability percentage was 92% ± 2.1% after 1 day, 81% ± 3.2% after 4 days, and 60.3% ± 2.3% after 7 days, while for the casted cell-laden hydrogel structure, the cell viability percentage was 94.5% ± 3.0% after 1 day, 83.9% ± 1.6% after 4 days, and 64.7% ± 2.5% after 7 days, which confirmed that the printing process is almost not damage to cells.

## Discussion

In general, the “printability” about a biomaterial contains at least three levels meaning. Firstly, the viscosity of this biomaterial should be adjustable, such as changing with temperature and shear thinning as the different printing methods may request different viscosity. Secondly, the biomaterial should be in liquid before printing to avoid nozzles jamming and solidification/gelatinization after printing as soon as possible to keep the shape. As a layer-by-layer method, gelatinization after printing is also important to glue the adjacent fibers. At last, finding the biofabrication window about the process parameters of a desired biomaterial is also very important, which determines whether this material can be widely used. After all, there are many promising materials which are not practical after the further printability investigation.

Currently most of researches about 3D bioprinting are focused on how to design new biomaterials^32^,^43^, how to vascularize inside the mimic organs^44^ and how to construct a suitable culture environment for organs functionalization^45^. However, in order to print structures of clinically relevant sizes, it is an additional important challenge to control the bioprinting fidelity and speed of biofabrication^32^. For example, aortic heart valve function is heavily dependent on its geometry^36^. Actually, some phenomena that affect the printing fidelity are easily observed in 3D bioprinting. Diffusion in the 3D grid pattern structure printing and the fidelity at central printed region was better than that at the edge region^36^.

In this report, we focused on giving a fabrication window of alginate/gelatin combinations about the printing parameters. This investigation can also help the assessment of other biomaterials. There are three purposes of adding gelatin during printing, including adjusting the viscosity, fixing the shape of printed structures and improving the cell adhesion and spreading. Viscosity of hydrogels is very important during printing, improper viscosity will lead to poor printed quality and even printing failure. As the viscosity of gelatin can also adjusted through concentration and temperature, it is a good additive for the improvement of printability and it can be used to enlarge the processing window.

In the view of fabrication, hydrogels usually have narrow windows for bioprinting. High concentrations/or crosslink densities are needed to keep good printing fidelity, which will limit the cell migration. However, Low concentrations usually have a poor printability. Also as cells must be mixed inside, many chemical or physical processes that usually used to enhance the strength and biocompatibility will not be affordable when the intermediate processes could damage the cells. It is still a big challenge to find an ideal printing biomaterial which has a suitable viscosity, enough strength, good biocompatibility & degradability.

## Conclusion

In this report, a series of experiments has been conducted to investigate the hydrogel printability. These results show that air pressure, feedrate and printing distance are the most important factors which can influence the printing quality. The printing resolution is also affected by the diffusion and fusion of the bioinks. It could be solved by reducing the extrusion rate or accelerating the moving speed. Through research of printing processing and systematical analysis of hydrogel structure, a set of appropriate printing process parameters are presented.

L929 mouse fibroblasts were encapsulated in hydrogel successfully and uniformly, and most of the cells were alive. Through contrast test, we demonstrated that the extrusion-based printing can almost have not extra damage to cells when comparing with casting. With good cell compatibility of the hydrogel material and the high printing quality with appropriate printing process parameters, it would be easy to precisely control the hydrogel deposition in the fabrication of mimic organ structures.

## Methods

### Hydrogel materials

Calcium chloride solution was prepared by dissolving CaCl_2_ power into deionized water to make the final concentration of 2% (w/v). All the printing material including the SA, gelatin, food dye and CaCl_2_ were purchased from Aladdin Industrial Corporation (Shanghai, China). The alginate/gelatin hydrogel was prepared as the following steps. Firstly, SA powder was dissolved into 95 ml deionized water with a magnetic stirrer for 4 h. Secondly, gelatin particles were added into the solution and the mixture was stirred 2 h in 37 °C water bath. Finally the hydrogel was stained by non-toxic red ink and it can be used for for printing. As the viscosity of SA is influenced by its concentration, different concentrations of mixture were prepared. The viscosity of mixture were investigated in 37 °C (in consideration of cell activity and avoiding nozzle jamming).

### Printability experiments

The printability experiments (1D structures printing, 2D structures printing and 3D structures printing) were proceeded with the same condition (nozzle heated up to 37 °C, substrate cooled to −5 °C). Mixture with 2.5% SA and 8% gelatin was set aside in 37 °C water bath for 4 h to 12 h to avoid air bubbles. In the air pressure experiment, the influence of *P* was determined via the distance from the separating location to the nozzle, and the morphological feature of printing lines were observed using a KEYENCE-VHX digital microscope. In the nozzle feedrate and printing distance experiments, line width was the indicator. In 2D and 3D structures printing experiments, the printability was judged by eyes.

### Cell culture and preparation of bioinks

Unless otherwise stated, L929 mouse fibroblasts were cultured in MEM with 10% fetal bovine serum, 1% penicillin and streptomycin in 37 °C, 5% CO_2_ environment. All of the regents were bought from Qizhenhu Biological Technology Co., Ltd., Hangzhou, China.

The cell-laden hydrogel ink was produced by adding L929 mouse fibroblasts into the hydrogel. Firstly, culture flasks with 95% cells confluency were took out from the incubator and washed with PBS (phosphate buffered saline). 0.25%. Trypsin-EDTA was added into culture flasks before they were put in the incubator (37 °C, 5% CO_2_) for 3 mins. Then the cell suspension was centrifuged at 1000 rpm for 5 mins. The supernatant was removed and the cells were resuspended in MEM cell culture medium to a concentration of 2 × 10^6^ cells/ml. The hydrogel consisted of 5% SA and 16% gelatin was prepared. At last, the hydrogel and the cells prepared as above were mixed at a volume ration of 1:1 and stirred for 5 min at 37 °C by a magnetic stirrer. And the final concentration of SA, gelatin and cell density was 2.5% (w/v), 8% (w/v) and 1 × 10^6^ cells/ml in bioinks.

In the control experiment, the alginate/gelatin hydrogel and the cells were prepared as mentioned before. The hydrogel and the cells were mixed at a volume ration of 1:1 and stirred for 5 min at 37 °C by a magnetic stirrer. And the final concentration of SA, gelatin and cell density was 2.5% (w/v), 8% (w/v) and 1 × 10^6^ cells/ml in bioinks. The casting mold in Fig. 14a was premade by a desktop 3D printer^46^. The cell-hydrogel was poured into the casting mold and put at 4 °C for 5 min in order to fix it. Then it was soaked in CaCl_2_ solution for 5 min. After crosslinking, the structure was took out by a pair of tweezers and washed by PBS for at least three times to remove residual CaCl_2_. Then it was put in a culture flask with right amount (the hydrogel can be immersed) of MEM with 10% fetal bovine serum, 1% penicillin and streptomycin. At last, the culture was put in the incubator (37 °C, 5% CO_2_).

### Viability analysis

When the 3D structures was printed with the cell-laden hydrogel ink, it was immersed into 2% CaCl_2_ solution for five minutes and then washed with PBS 3–5 times to remove the residual CaCl_2_. The 3D structure was culture in petri dish containing MEM with a condition of 37 °C, 5% CO_2_. After 1d/4d/7d, the cell viability of the 3D structure was examined using a Calein AM-PI mixture (KeyGEN BioTECH Co., Ltd., Nanjing, China) for 30 min in the dark and washed three times with PBS. According to the fluorescent images, red indicated dead cells and green indicated live cells. The images were acquired using a laser scanning confocal microscope. Cell viability was calculated as (number of live green stained cells/number of total cells) × 100%. Three independent samples were counted and each sample was taken eight pictures uniformly to be observed under LSCM (LSM780, ZEISS). The number of green or red cells were quantified with ImageJ’s cell counter.

## Additional Information

**How to cite this article**: He, Y. *et al*. Research on the printability of hydrogels in 3D bioprinting. *Sci. Rep.* **6**, 29977; doi: 10.1038/srep29977 (2016).

## Supplementary Material

Supplementary Information

## Figures and Tables

**Table 1:** Viscosity of sodium alginate (37 °C, Concentration, %w/v).

C	**Max of η/cps**	**Min of η/cps**	**Average of η/cps**	**ln** ***η***	
1.0%	90	85	89	4.49	
1.5%	273	268	271	5.60	
2.0%	575	560	568	6.34	
2.5%	1.69 × 10^3^	1.63 × 10^3^	1.66 × 10^3^	7.41	
3.0%	2.29 × 10^3^	2.26 × 10^3^	2.27 × 10^3^	7.73	
3.5%	4.85 × 10^3^	4.60 × 10^3^	4.68 × 10^3^	8.45	
4.0%	1.06 × 10^4^	1.04 × 10^4^	1.04 × 10^4^	9.25	
4.5%	2.79 × 10^4^	2.45 × 10^4^	2.68 × 10^4^	10.20	
5.0%	3.58 × 10^4^	3.4 × 10^4^	3.47 × 10^4^	10.45	

**Table 2:** Viscosity (ln *η*) of the mixture of sodium alginate and gelatin (37 °C, Concentration, %w/v).

*C*/gelatin	*C*/Soduim alginate				
	**1%**	**2%**	**2.5%**	**3%**	**4%**
2%	4.93	7.10	7.81	8.45	9.51
4%	5.37	7.59	8.42	8.68	9.74
6%	6.08	7.82	8.57	8.98	10.11
8%	6.50	8.12	8.85	9.39	10.43
10%	6.72	8.68	9.19	9.69	10.60
