# Supplementary material for: Research on the printability of hydrogels in 3D bioprinting
Source: Sci Rep. 2016 Jul 20;6:29977. doi: 10.1038/srep29977 (PMC4951698; doi:10.1038/srep29977)
Supplement: Supplementary Information [file srep29977-s1.pdf]

Supplemental Materials

Research on the printability of hydrogels in 3D bioprinting

Yong He[1,2,3]*, FeiFei Yang[1,2], HaiMing Zhao[1,2], Qing Gao[1,2], Bing Xia[1,2], JianZhong Fu[1,2*]

(1. State Key Laboratory of Fluid Power and Mechatronic Systems, College of Mechanical Engineering, Zhejiang University, Hangzhou 310027, China

2. Key Laboratory of 3D Printing Process and Equipment of Zhejiang Province, College of Mechanical Engineering, Zhejiang University, Hangzhou 310027, China

3. State Key Laboratory for Manufacturing Systems Engineering, Xi'an Jiaotong University, 710054, Xi'an China

**Correspondence to: Yong He; e-mail: yongqin@zju.edu.cn or JianZhong Fu; e-mail: fjz@zju.edu.cn)*

(a)

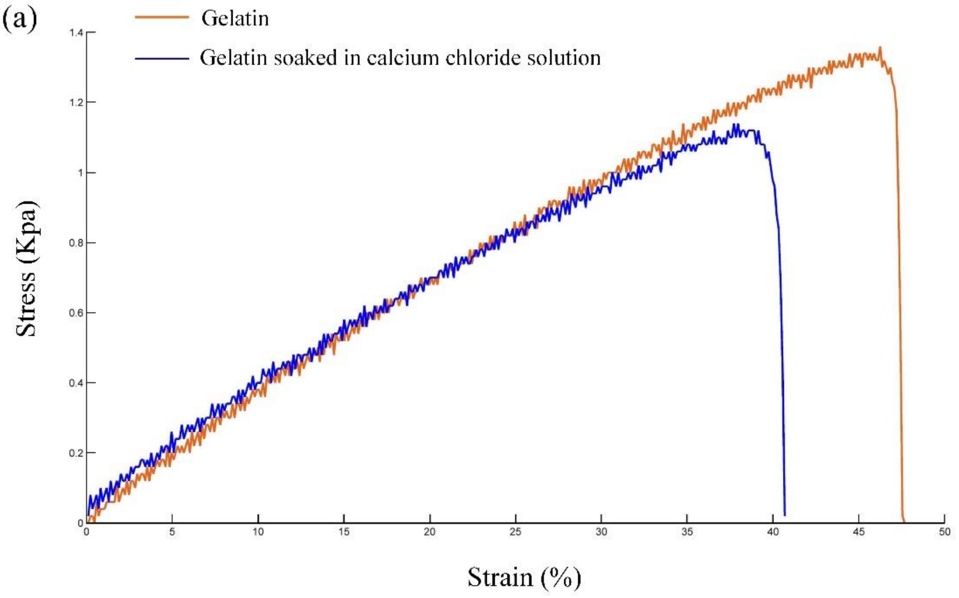

(b)

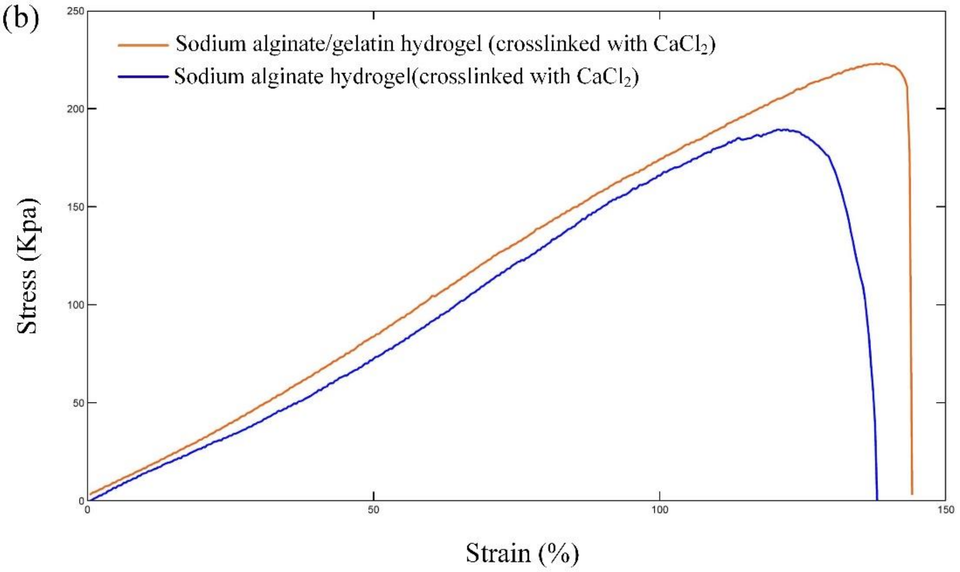

Fig.S1 Stress-strain curves of different materials. (a) Stress-strain curves of gelatin and gelatin soaked in calcium chloride solution. (b) Stress-strain curves of sodium alginate/gelatin hydrogel (crosslinked with CaCl2) and sodium alginate hydrogel (crosslinked with CaCl2).

Tensile strength of different materials shown in Fig.S1 was performed by making uniaxial measurements using tension gauge DS-5N (ZHIQU, CHINA) at room temperature. Gelatin gels were cut into bone-shape specimens with a cross-section area of 3.5×1.5 mm after they were took from the environment of 4°C immediately. They were clamped horizontally, with a gauge length of 25mm and tested at a constant rate of 1.33 mm/s. All the samples were stretched until failure. Stress was calculated by dividing the force generated during extension by the initial cross-sectional area. While the sodium alginate hydrogel and sodium alginate/gelatin hydrogel were cut into bone-shape specimens with a cross-section area of 3.5×1.5 mm after they were crosslinked with $CaCl_2$ at room temperature.

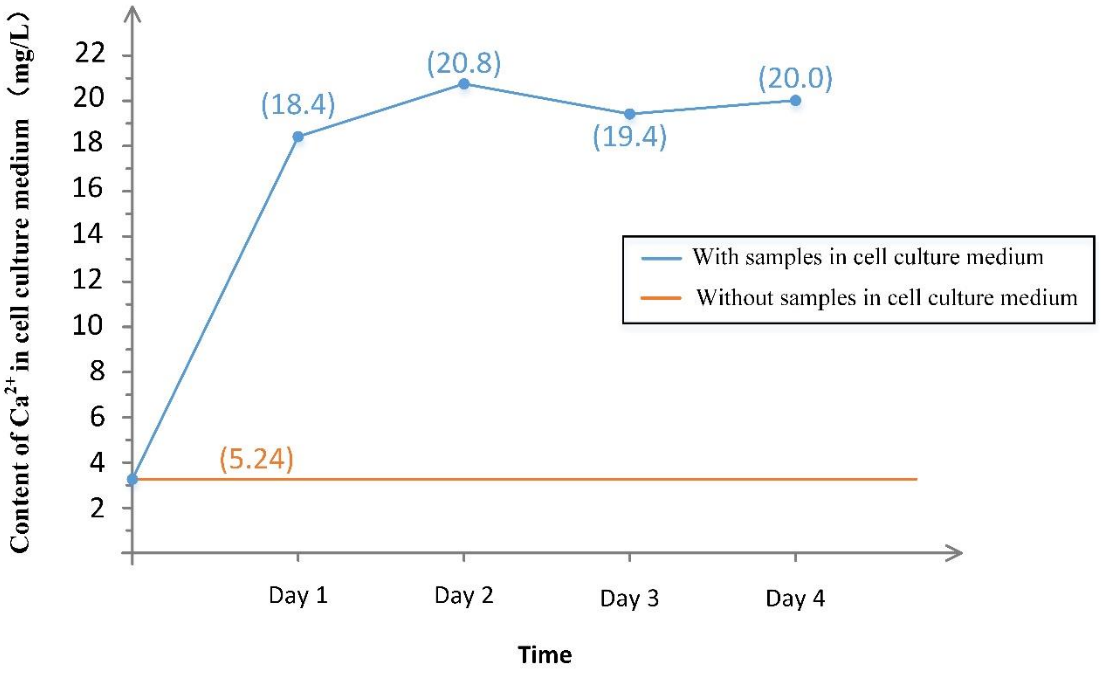

Fig.S2 The content of Ca^{2+} in cell culture medium in 4 days.

The experiment of concentration of calcium ion (Ca^{2+}) shown in Fig.S2 were proceeded as follows. Mixture with 2.5% SA and 8% gelatin was prepared as described in the paper. The structures and the creation method of the samples were in the same as in the paper (Fig.13b). Then the samples were cultured in 10ml MEM with 10% fetal bovine serum, 1% penicillin and streptomycin in 37°C, 5% CO_2 environment. The cell culture medium without samples in it was prepared as the control group. The result of the experiment was obtained using Atomic Absorption Spectrometer HITACHI 180-50 as shown in Fig.4. We found the content of Ca^{2+} in cell culture medium increased a lot after 24h. However, it was increased very few and became stable at the concentration of 20mg/L in the following three days.
